# Intrafamilial phenotypic heterogeneity in a Taiwanese family with a *MAPT* p.R5H mutation: a case report and literature review

**DOI:** 10.1186/s12883-017-0966-3

**Published:** 2017-09-18

**Authors:** Hui-Chi Lin, Chin-Hsien Lin, Pei-Lung Chen, Shih-Jung Cheng, Pei-Hao Chen

**Affiliations:** 1 Department of Neurology, MacKay Memorial Hospital, No. 92, Sec. 2, Zhongshan N. Rd., Zhongshan Dist, Taipei City, 10449 Taiwan; 2 Department of Neurology, National Taiwan University Hospital, No. 7, Chung-Shan South Road, Taipei, 100 Taiwan; 3 Department of Medical Genetics, National Taiwan University Hospital, Taipei, Taiwan; 4 Graduate Institute of Medical Genomics and Proteomics, National Taiwan University College of Medicine, No. 7, Chung-Shan South Road, Taipei, Taiwan; 5 Department of Medicine, Mackay Medical College, New Taipei, Taiwan; 6 Graduate Institute of Mechanical and Electrical Engineering, National Taipei University of Technology, Taipei, Taiwan

**Keywords:** Frontotemporal dementia, Primary progressive aphasia, Microtubule-associated protein tau, Amyotrophic lateral sclerosis, Corticobasal syndrome, Depression

## Abstract

**Background:**

Frontotemporal degeneration (FTD) is a clinically and genetically heterogeneous neurodegenerative disorder characterized by deficits in executive function that frequently overlaps with parkinsonism and motor neuron disorders. Several genes have been identified to cause autosomal dominant forms of FTD, including the gene coding for the protein associated with microtubule tau (*MAPT*). While most reported pathogenic mutations in MAPT occur in exons 9–13, few families have been reported with mutations outside of this region. Herein, we report a first Taiwanese family having the exon 1 p.Arg5His mutation in *MAPT* with intrafamilial phenotype heterogeneity.

**Case presentation:**

A 63-year-old man presented with progressive non-fluent speech and impaired memory for 3 years. He then developed apraxia, myoclonus and parkinsonism feature at his right hand. Extensive neurologic and neurocognitive examination lead to a diagnosis of FTD mixed with corticobasal syndrome. Magnetic resonance imaging revealed asymmetric atrophy in the left frontal and temporal lobes and single-photon emission computed tomography indicated decreased metabolism in the same areas as well as the left basal ganglia. The patient’s mother had been diagnosed with amyotrophic lateral sclerosis (ALS) at the age of 60 and was deceased 10 years later due to respiratory failure. The patient’s younger sister had persistent depressive disorder in her early forties and did not have any prominent cognitive or motor dysfunctions. We performed genetic analysis applying a targeted next generation sequencing (NGS) panel covering *MAPT*, *GRN*, *VCP*, *FUS*, *CHMP2B*, and *TARDBP* on the proband, followed by Sanger sequencing of candidate genes in eight family members. Hexanucleotide repeat expansion of *C9Orf72* was determined by repeat-primed PCR. We identified a missense mutation in exon 1 of *MAPT* gene, c.14G > A (p.R5H), which was previously reported in only two Japanese patients in a literature review. This substitution co-segregated with the disease phenotypes in the family.

**Conclusions:**

This is the first report of the occurrence of the *MAPT* p.R5H mutation in the Taiwanese population. Our findings extend the current knowledge of phenotypic heterogeneity among family members carrying the *MAPT* p.R5H mutation.

## Background

Frontotemporal dementia (FTD) is a heterogeneous neurodegenerative disorder characterized by changes in personality, executive function deficits and language impairment, which symptoms are often co-morbid with parkinsonism features and motor neuron disorders [1]. FTD is the second most common cause of dementia in people under 65 years of age and contributes to 5–15% of patients with dementia [2]. FTD prevalence is 15 to 22 per 100,000 people in the 45–64 year age range, and the incidence rate is between 2.7 and 4.0 per 100,000 person-years [3]. The clinical presentations of FTD can be divided into a behavioral variant of FTD (bvFTD) and primary progressive aphasia (PPA). PPA can be further divided into semantic dementia, logopenic progressive aphasia and progressive non-fluent aphasia [1]. Compared with Alzheimer’s disease, FTD is more commonly associated with parkinsonism and in some cases with motor neuron disease as well, particularly amyotrophic lateral sclerosis (ALS) [4]. Parkinsonism is usually observed in patients with bvFTD, but is rarely seen in those with other types of FTD [5].

A positive family history in patients with FTD has been identified in 30–50% of cases, and 10–20% of these have an autosomal dominant pattern of inheritance [1]. Mutations in the *MAPT*, *GRN*, and *C9orf72* genes account for approximately 17% of cases among all forms of FTD, with *GRN* and *MAPT* accounting for approximately 5%–20% of all familial FTLD cases [1, 6]. Post-mortem pathology findings also reflect the heterogeneity of FTD. The underlying pathological changes observed in patients with FTD are classified based on the constituents of the intra-neuronal protein aggregations, including tau, tau-negative but ubiquitin-positive inclusions, transactive response DNA-binding protein 43 kDa (TDP-43), and fused-in-sarcoma protein (FUS) [7]. Among the genes known to cause autosomal dominant forms of FTD, *MAPT* mutations are identified in up to 20% of familial forms of FTD [8, 9] and at least 50 *MAPT* gene mutations have been identified in individuals from more than 100 families with FTD [10, 11]. Most *MAPT* mutations are found in exons 9–13, which encode the microtubule binding domains that mediate interaction of tau with microtubules, as well as flanking regions. Few mutations outside exons 9–13 have been reported [12, 13].

Here we report the results of a genetic study applying targeted next generation sequencing (NGS) in a Taiwanese family with the exon 1 p.Arg5His mutation in *MAPT* and intrafamilial phenotype heterogeneity, including symptoms of FTD, corticobasal degeneration (CBD), ALS, and persistent depressive disorder (PDD).

## Case presentation

**Fig. 1:**
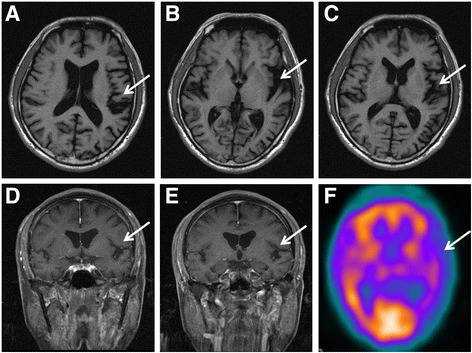
Brain MRI of the index patient. **a**-**e** T1WI MRI series showed focal atrophy in the posterior frontal premotor cortex (**a**-**c**, arrows) and superior temporal gyrus (**d**, **e**, arrows). **f** SPECT revealed decreased metabolism in left temporal and occipital cortex (arrows)

A 63-year-old right-handed man, with underlying well controlled arterial hypertension and hyperlipidemia, presented with verbal expression difficulty and non-fluent speech that had been progressing for 3 years. He also had difficulty with word finding and naming, stuttering, and verbal hesitancy. Several months later, he found that he was unable to use chopsticks well and could not perform delicate movements using his right hand. Neurological examinations indicated the presence of mask face, right-hand apraxia, bradykinesia, and rigidity, as well as stimulus-sensitive myoclonus on his right hand and forearm. No personality changes, behavioral disturbances, psychiatric symptoms, limited eye movement, bulbar symptoms, retrocollis or antecollis, respiratory distress, focal weakness, focal numbness, or gait disturbances were observed. The deep tendon reflex and cerebellar and sensory systems were all normal. He scored 23/30 on the mini-mental state examination, with impairments in the attention, short-term memory, language, and visuospatial domains [14]. A complete neuropsychological and cognition examination revealed impaired attention, transcortical motor aphasia, impaired verbal memory, impaired visuospatial abilities, and executive dysfunction. Laboratory tests for secondary dementia found negative results of syphilis screening, and the results of serum vitamin B12, folic acid, and thyroid function were all within normal limits. Brain magnetic resonance imaging (MRI) showed asymmetric atrophy of the left frontal and temporal lobes (Fig. 1a-e), and single-photon emission computed tomography (SPECT) revealed decreased metabolism in the left temporal and low parietal cortices (Fig. 1f). These findings indicated a clinical diagnosis of the PPA variant of FTD, combined with CBD features over his right limbs. We prescribed donepezil, and the patient’s cognition stabilized over the following year. We were unable to assess levels of total and phospho-tau proteins in the cerebrospinal fluid due to the patient’s unwillingness to receive a lumbar puncture examination.

**Fig. 2:**
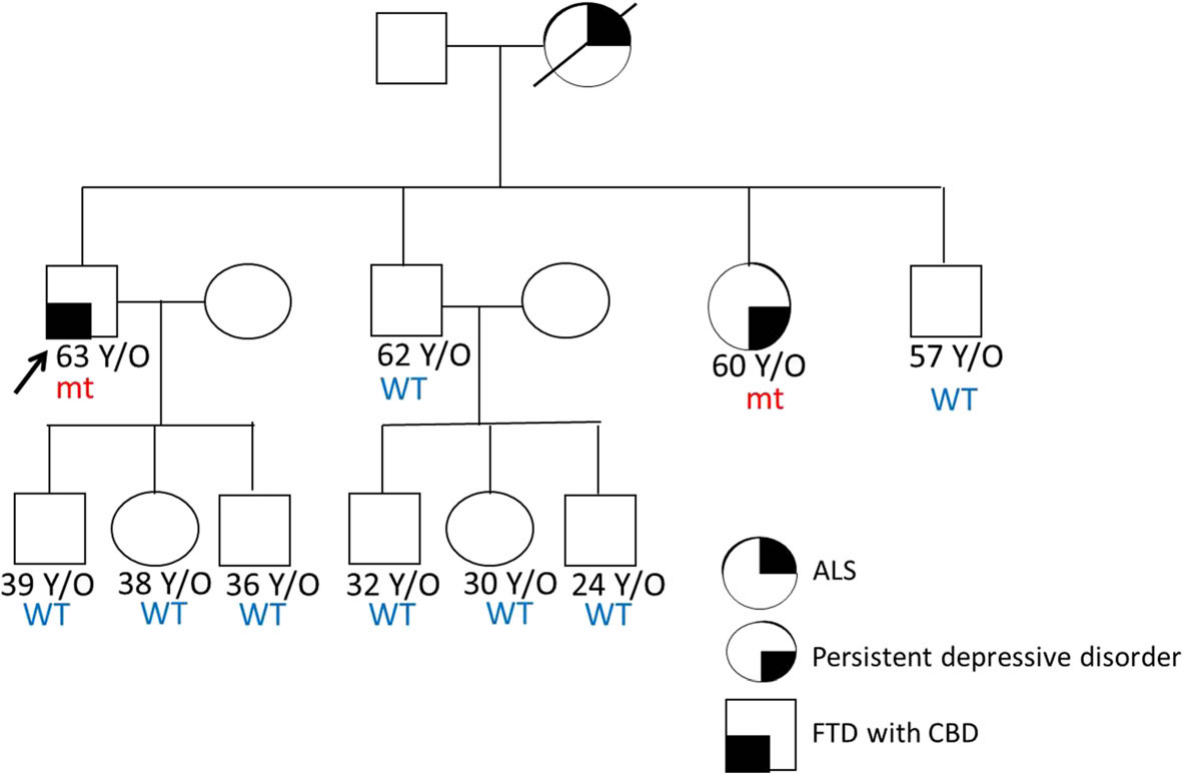
Family pedigree and genetic analysis of the index patient. Index family pedigree. Black symbols denote family members affected with FTD, ALS, or PDD. The proband described in the current study is marked with an arrow. mt, mutated alleles; wt, normal alleles

The patient’s family history showed that his deceased mother and younger sister both had neuropsychiatric disorders (Fig. 2). The patient’s mother had been diagnosed with ALS at age 60 (Fig. 2), and the patient’s sister was known to have PDD, although she declined additional detailed cognition examinations (Fig. 2). Neurological examinations of other family members, including additional siblings and the younger generation, were all normal.

Next generation sequencing (NGS) allows for the comprehensive genetic analysis of familial neurodegenerative syndromes, as seen in FTD with parkinsonism [1]. Since the clinical picture in this case indicated an autosomal dominant inheritance form of FTD with neuropsychiatric disorder, we applied genetic analysis using targeted NGS and covering candidate genes known to cause familial forms of FTD and ALS to the proband [15]. We extracted variants within the genes of interest for further analysis, including *MAPT* (NM_001123066.3), *GRN* (NM_002087.2), *FUS* (NM_001170937), *TARDBP* (NM_007375), *VCP* (NM_007126) and *CHMP2B* (NM_014043). Abnormal GGGGCC hexanucleotide repeat expansion in the *C9Orf72* (NM_018325) gene was determined by repeat-primed PCR, which was carried out as previously described [16]. Fragment length analysis and Sanger sequencing were performed using an ABI 3730xl genetic analyzer (Applied Biosystems, Foster City, CA, USA). The frequency of the identified variants in the general population were checked using the dbSNP, Exome Aggregation Consortium (ExAC), 1000 Genomes Project, and Taiwan Biobank (https://www.twbiobank.org.tw/new_web/index.php), a whole genome sequencing (WGS) database enrolling 997 Taiwanese people. The functional annotation of the variants was determined by the prediction software to obtain a prediction of pathogenicity. We considered variants potentially pathogenic if they had a minor allele frequency (MAF) < = 0.05%, or were predicted to change the amino acid sequence or the splicing junction. Mutations were defined as pathogenic if they had been previously reported in the literature as a causative variant, or if the pathogenicity was confirmed by segregation analysis.

**Fig. 3:**
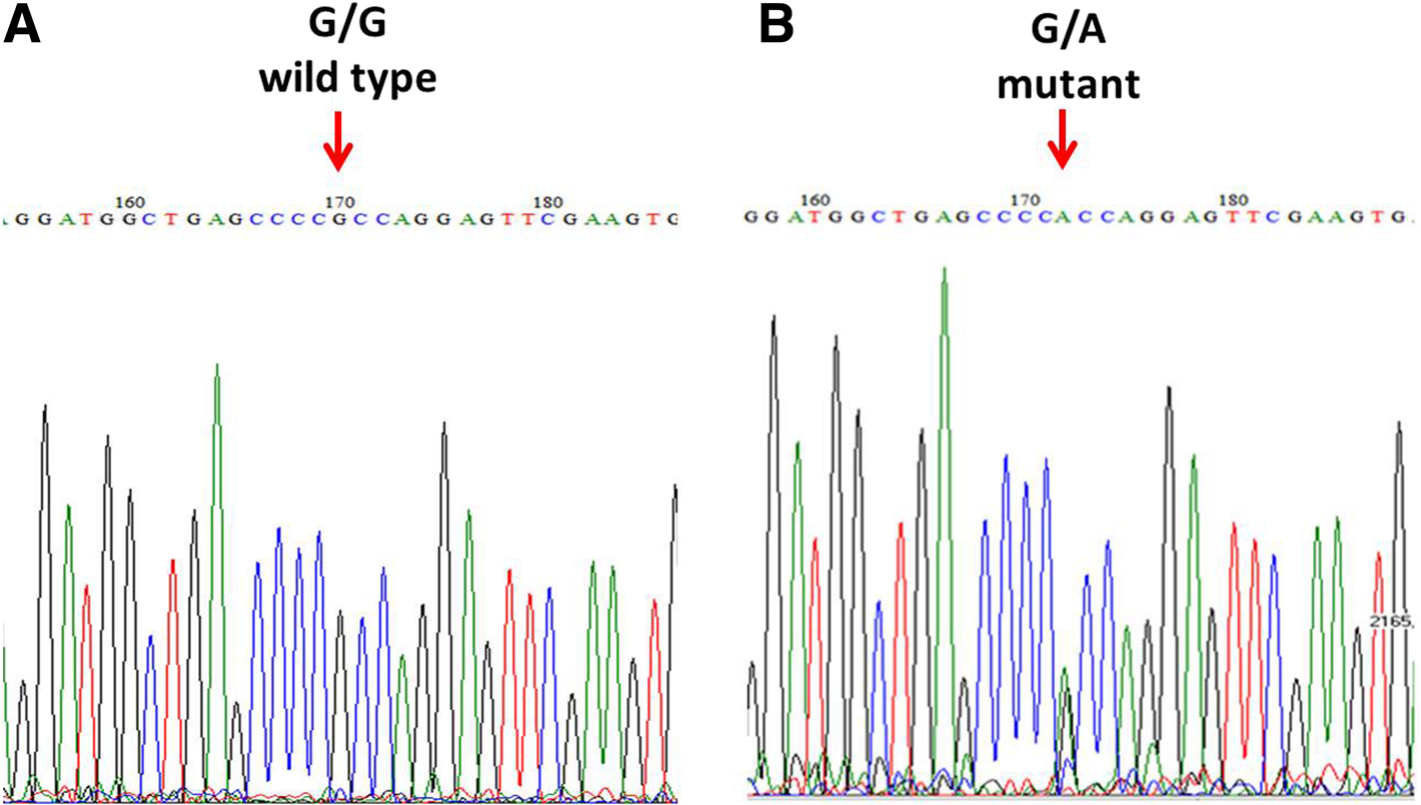
Chromatograms of direct sequencing of the *MAPT* genomic sequence. Genetic analysis revealed a single nucleotide change (c.14G > A, p.R5H, right panel) compared to the wild type sequence (left panel). The mutations identified in this study were located in the indicated position

We identified a heterozygous missense substitution in exon 1 of the *MAPT* gene in this patient, c.14G > A (p.R5H) (Fig. 3). In silico, PolyPhen2 predicted that this substitution was likely damaging. This genetic variation had also been reported in previous studies as the genetic cause of two familial FTD cases, and was absent in more than 100 Japanese control subjects [12, 13, 17]. This genetic substitution co-segregated with the disease phenotype in the patient’s family (Fig. 2) and is a rare variant in both the general and the Taiwanese populations, found in less than 1/10000 in the ExAC and absent from the WGS database of the Taiwan Biobank. This study was approved by the institutional ethics committee of National Taiwan University Hospital, and written informed consent was obtained from all subjects from whom blood samples were obtained for genetic testing.

## Discussion and conclusions

To date, most reported mutations identified in the *MAPT* gene are located in exons 9–13 [10, 11], and few mutations outside of those exons have been reported. Here, we present the first Taiwanese family with a pathogenic variant in exon 1 of the *MAPT* gene, c.14G > A (p.R5H), causing a heterogeneous neurological phenotype including the PPA variant of FTD, CBD, ALS and PDD.

Alternate splicing of exons 2, 3, and 10 of *MAPT* generates six tau isoforms with 3 or 4 amino-acid repeats (3R or 4R) [18]. Since the p.P301L mutation in the *MAPT* gene was first described in several families with FTD and parkinsonism in 1998 [19, 20], more than 50 mutations have been identified and are located in exons 9–13, known to encode the microtubule binding domains (mediating the interaction of tau and microtubules) and intronic mutations affecting exon 10 splicing, that then produce either 4R tau or 3R tau [5, 9]. These mutations exert their effect through a toxic gain of function mechanism, either by reducing the ability of tau to interact with microtubules or by affecting the splicing of exon 10 [21]. Mutations outside exons 9–13, especially in exon 1, are rare. A single case of FTD and Parkinsonism with the exon 1 p.R5H variant was reported by Hayashi et al. [13] and Poorkaj et al. reported a single case of PSP with the p.R5L variant [12]. Recently, three persons with dementia including one with ALS in a two-generation family were identified to have the *MAPT* p.R5H substitution [17]. The *MAPT* p.D348G mutation was found in a large Italian family with pure lower motor neuron degeneration, implicating defects in the tau degradation pathway in motor neuron degeneration [22]. Interestingly, the case presented in Hayashi et al. was Japanese, and the family reported by Leverenz et al. is Japanese-American [13, 17]. Since this substitution did not appear in the WGS data from 997 Taiwanese subjects and it was also not found in more than 100 control Japanese subjects in previous studies, we can conclude that this variant is not a common polymorphism in the Asian population [12, 13, 17]. Postmortem analysis of one PSP patient with a *MAPT* p.R5H substitution revealed neuronal loss in the frontal and temporal lobes as well as widespread tau deposits in both neurons and glia [13]. Extensive tau filament depositions composed of 4R tau were also observed [13]. This mutation reduces the ability of tau to promote microtubule assembly and is known to promote fibril formation in vitro [13].

Previous studies have shown that FTD, CBS, PSP, and motor neuron disorders can be regarded as a clinically and biologically cohesive spectrum of tauopathy with neuronal inclusions exhibiting distinct isoforms of tau [1], and the clinical association of FTD and motor neuron disorders occurs in approximately 5% of FTD cases [23]. However, cognitive dysfunction is also observed in patients with ALS, and may reflect abnormal tau protein metabolism [24–26]. Previous studies in both Caucasian and Asian populations have shown that a *MAPT* gene polymorphism is strongly associated with sporadic ALS [27, 28]. Although ALS has been reported in patients carrying the *MAPT* gene mutation p.K317 M [29], this phenotype was not reported in patients carrying the *MAPT* p.R5H mutation. Furthermore, most carriers of the *MAPT* p.R5H mutation phenotypes present as FTD combined with features of PSP-like parkinsonism (Table 1). In this case, our index patient presented with CBS-like symptoms and the PPA variant of FTD, and the mother of the index patient was diagnosed with ALS, extending the current knowledge of the phenotypes associated with this rare substitution in *MAPT* (Table 1). These observations support the marked phenotypic heterogeneity seen among members of the same family carrying the same *MAPT* mutations.

**Table 1:** Clinical phenotypes of patients carrying *MAPT* exon 1 mutation

Subjects/reference	Age of onset (years, range)	FTD	Motor neuron disease	Parkinsonism	Symptoms of other family members	Ethnicity	Genetic findings
Hayashi S et al., 2003 [13]	76	+ (no specific sub-type was described)	–	+ PSP	Late-onset dementia	Japanese	c.14G > A (p.R5H)
Leverenz J et al., 2011 [17]	72–86	+ (no specific sub-type was described)	–	–	Late-onset dementia	Japanese-American	c.14G > A (p.R5H) (with pathology proof)
Poorkaj P et al., 2002 [12]	N.A.	–	–	+ PSP	N.A.	N.A.	c.14G > T (p.R5L)
Index patient of the current study	60	+ (PPA variant of FTD)	–	+ CBD	Motor neuron disorder (ALS)	Taiwanese	c.14G > A (p.R5H)

*PPA* primary progressive aphasia, *PSP* progressive supranuclear palsy, *CBD* corticobasal degeneration, *ALS* amyotrophic lateral sclerosis, *N.A.* not available

PDD is one of the phenotypes observed in a *MAPT* p.R5H carrier in this index family. FTD onset can resemble or be misdiagnosed as various different psychiatric disorders, including depression, bipolar, obsessive compulsive, and impulse-control disorders [30–32]. Psychiatric features can vary depending on the regions that are involved early in the disease process, and early emotional disturbances can be present in patients with FTD that primarily affects the temporal lobes [33]. Therefore, the initially presenting symptoms of FTD could be depression, psychosis, compulsions, anxiety, agitation, or other psychiatric features that are not typically included in the behavioral criteria for this disorder [33–35]. Overlapping presentation between depression and personality change could mask the true disease, especially early in the disease course [36]. Depression was significantly more common in the semantic dementia variant of FTD than in bvFTD [37]. Pre-existing psychiatric disorders can also evolve after decades of illness and be comorbid with FTD. A previous study reported a female patient with FTD who presented with major depressive disorder, which contributed to the delay of proper diagnosis and treatment for FTD [38]. A second study also reported bipolar affective disorder as the initial presentation of a patient ultimately diagnosed with FTD [31, 39]. Microtubule-related cytoskeleton dysfunction has been inferred in the mechanisms underlying the pathology of several neuropsychiatric disorders, including major depression and bipolar affective disorders [40, 41]. These observations reinforce the finding that microtubule instability caused by *MAPT* mutations contributes to both neurodegenerative and psychiatric disorders. Depressive symptoms, especially major depressive disorder, should be evaluated cautiously in patients with FTD.

This report has several strengths, defined by the fact that it is the first Taiwanese family reported with a pathogenic variant in *MAPT* and variable phenotypes across several family members. Moreover, phenotypes including CBD and PDD add evidence for the variable spectrum associated with *MAPT* p.R5H mutation. The identification and report of families with a *MAPT* mutation provide the opportunity to investigate potential biomarkers in very early stages of the disease, such as 14hosphor-tau expression levels and total tau in cerebrospinal fluid and plasma, in carriers of the mutation. However, there are also some limitations to the current study, including the lack of pathological confirmation of the diagnosis of the index patient as the PPA variant of FTD, and the limited number of enrolled family members.

In summary, we present a report of the first Taiwanese family presenting with the PPA variant of FTD, CBD, ALS, and PDD and carrying a *MAPT* p.R5H mutation. These findings highlight the marked phenotypic variability possible within subjects carrying the same *MAPT* mutation, and specifically extend the current knowledge of phenotypes associated with the *MAPT* p.R5H mutation.

## Abbreviations

FTD: frontotemporal dementia
MAPT: microtubule-associated protein tau
